# Reference blood pressure values obtained using the auscultation method for 2-year-old Japanese children: from the Japan Environment and Children’s Study

**DOI:** 10.1007/s10157-023-02370-w

**Published:** 2023-06-30

**Authors:** Naoya Fujita, Hidetoshi Mezawa, Kyongsun Pak, Osamu Uemura, Kiwako Yamamoto-Hanada, Miori Sato, Mayako Saito-Abe, Yumiko Miyaji, Limin Yang, Minaho Nishizato, Yukihiro Ohya, Kenji Ishikura, Yuko Hamasaki, Tomoyuki Sakai, Kazuna Yamamoto, Shuichi Ito, Masataka Honda, Yoshimitsu Gotoh, the Japan Environment, Children’s Study Group, Michihiro Kamijima, Shin Yamazaki, Reiko Kishi, Nobuo Yaegashi, Koichi Hashimoto, Chisato Mori, Zentaro Yamagata, Hidekuni Inadera, Takeo Nakayama, Tomotaka Sobue, Masayuki Shima, Hiroshige Nakamura, Narufumi Suganuma, Koichi Kusuhara, Takahiko Katoh

**Affiliations:** 1 Department of Pediatric Nephrology, Aichi Children’s Health and Medical Center, 4267-Chome, Morioka-cho, Obu, Aichi 474-8710 Japan; 2 Medical Support Center for Japan Environment and Children’s Study, National Center for Child Health and Development, 2-10-1 Okura, Setagaya-ku, Tokyo, 157-8535 Japan; 3 Division of Biostatistics, Department of Data Management, Center for Clinical Research and Development, National Center for Child Health and Development, 2-10-1 Okura, Setagaya-ku, Tokyo, 157-8535 Japan; 4 Department of Pediatrics, Ichinomiya Medical Treatment & Habilitation Center, 1679-2 Tomida-nagaresuji, Ichinomiya-city, Aichi 494-0018 Japan; 5 Department of Pediatrics, Kitasato University School of Medicine, 1-15-1 Kitazato, Minami-ku, Sagamihara, Kanagawa 252-0374 Japan; 6 Department of Nephrology, Faculty of Medicine, Toho University, 6-11-1 Omori Nishi, Ota-ku, Tokyo, 143-8541 Japan; 7 Department of Pediatrics, Shiga University of Medical Science, Tsukinowa, Otsu, Shiga 520-2192 Japan; 8 Department of Pediatrics, Graduate School of Medicine, Yokohama City University, 3-9 Fukuura, Kanazawa-ku, Yokohama, 236-0004 Japan; 9 Department of Pediatric Nephrology, Tokyo Metropolitan Children’s Medical Center, 2-8-29 Musashidai, Fuchu, Tokyo 183-8561 Japan; 10 Department of Pediatric Nephrology, Japanese Red Cross Aichi Medical Center Nagoya Daini Hospital, 2‐9 Myoken‐cho, Showa‐ku Nagoya‐shi, Aichi, 466‐8650 Japan

**Keywords:** Blood pressure, Reference value, Auscultation, Aneroid sphygmomanometer, LMS method, Polynomial regression model

## Abstract

**Background:**

Reference blood pressure (BP) values for Japanese children based on a large number of measurements by auscultation have not yet been established.

**Methods:**

This was a cross-sectional analysis of data from a birth-cohort study. The data from the sub-cohort study conducted for children at the age of 2 years in the Japan Environment and Children’s Study from April 2015 to January 2017 were analyzed. BP was measured via auscultation using an aneroid sphygmomanometer. Each participant was measured in triplicate, and the average value of two consecutive measurements with a difference of less than 5 mmHg was recorded. The reference BP values were estimated using the lambda–mu–sigma (LMS) method and compared with those obtained via the polynomial regression model.

**Results:**

Data from 3361 participants were analyzed. Although the difference between the estimated BP values by the LMS and the polynomial regression model was small, the LMS model was more valid based on the results of the fit curve of the observed values and regression models for each model. For 2-year-old children with heights in the 50th percentile, the 50th, 90th, 95th, and 99th percentile reference values of systolic BP (mmHg) for boys were 91, 102, 106, and 112, and that for girls were 90, 101, 103, and 109, respectively, and those of diastolic BP for boys were 52, 62, 65, and 71, and that for girls were 52, 62, 65, and 71, respectively.

**Conclusion:**

The reference BP values for 2-year-old Japanese children were determined based on auscultation and were made available.

**Supplementary Information:**

The online version contains supplementary material available at 10.1007/s10157-023-02370-w.

## Introduction

Children with hypertension (HT) account for approximately 3% of the pediatric population [1, 2], but some studies have shown an increase in the number of these individuals [3], which is of concern [4]. Previous reports have shown that childhood HT can cause target organ damage, such as cardiovascular disease [5] in adulthood [6] and may increase the risk of atherosclerosis [7]. However, some studies have reported that blood pressure (BP) reduction can reduce the risk of target organ damage [8, 9]. Therefore, BP control during childhood is essential.

Appropriate reference values are required for proper BP management; the following factors should be considered when designating reference values for BP: first, an auscultatory method should be used to measure BP. BP obtained via aneroid manometers is comparable to that via mercury sphygmomanometers, while SBP measured with oscillometric devices are slightly higher than that measured with mercury sphygmomanometers [10]. Although oscillometric devices are widely used in BP screening in children, HT should be diagnosed by auscultation using the reference values [11]. The reference values for BP in children that are widely used in the United States and Europe were established via auscultation [11, 12]. Second, the reference value for BP should be appropriate for each race or ethnicity. Racial differences reportedly occur in BP, even in children [2]. Previous reports have introduced auscultatory BP reference values for children in Western [13–15] and other Asian countries [16]; however, no such reference values have been identified for Japanese children. Currently, the most widely used BP reference values in Japan are those from the US [13]. However, their applicability in Japanese children is insufficiently verified. Therefore, it is necessary to establish auscultatory BP reference values for Japanese children.

The Japan Environment and Children’s Study (JECS) is a nation-wide birth cohort study to discern the effects of fetal and childhood environmental factors on the health and development of children [17, 18]. This study began in 2010 and will survey approximately 100,000 pregnant women and their offspring at 15 Regional Centers and 300 Co-operating health care providers across Japan and will continue until the children reach the age of thirteen. In addition to surveying participants, the JECS plans detailed surveys of approximately 5000 children, named the Sub-Cohort Study, including auscultatory BP measurement at the 15 Regional Centers at the ages of 2, 4, 6, 8, 10, and 12 years. To date, only the detailed survey for 2-year-olds has been completed.

This study establishes reference BP values from the Sub-Cohort Study for 2-year-old Japanese children using BP measurements obtained via the auscultatory method.

## Methods

### Study design, study participants, and data collection

This was a cross-sectional analysis of data from the Sub-Cohort Study. The details of the JECS main study and the Sub-Cohort Study have been described elsewhere [18, 19] (Supplementary Method). Of the 100,303 children in the JECS main study, 5015 participated in the Sub-Cohort Study. The participants who met the eligibility criteria of the Sub-Cohort Study were randomly recruited. The present study was based on the dataset “jecs-ta-20190930”, released in October 2019.

We excluded children of the Sub-Cohort Study with: (1) chronic diseases; (2) two or more missing BP values or a diastolic BP (DBP) value of 0 mmHg or not applicable; (3) unstable posture or condition for measuring BP, such as children unable to maintain a sitting or supine position, those crying or sleeping during measurement, or those with a fever; (4) unstable BP values with a difference of ≥ 5 mmHg for two consecutive BP measurements; (5) either missing covariates or outliers for height measurements; (6) children under 24 months of age.

### BP measurement procedure and BP value selection

The standardized BP measurement in the Sub-Cohort Study is shown in Supplementary Table S1. This method complied with the American Academy of Pediatrics (AAP) guidelines [11], and the doctors or nurses took all BP measurements. Each participant was measured thrice to ensure stable measurement value according to the BP definition in previous major BP management guidelines [11, 12, 20, 21]. BP measuring postures and conditions are collected simultaneously. The Sub-Cohort Study used aneroid sphygmomanometers (DS66 DuraShockTM hand aneroid [Welch Allyn Inc, Syracuse, NY, USA]). Each sphygmomanometer was visually inspected every test day before the investigation, and the investigator repaired or replaced it as required. Although the accuracy guarantee by the manufacturer was ten years, JECS strictly operated them within the accuracy guarantee period and replaced them every 4 years.

We adopted the BP value when comparing the average of two consecutive measurements with a difference of < 5 mmHg. The difference among the three measurements was < 5 mmHg, and the average of the second and third BP values was adopted. Moreover, when the second BP measurement was missed, we adopted the average of the other two when the difference between the first and the third one was < 5 mmHg.

### Covariates

Data on the sex, gestational weeks, birth weight, and diagnosis of chronic disease in the children were collected from the JECS main study. Diagnosis of chronic disease was defined based on medical record transcripts at birth and one month of age and parent-reported doctor-diagnosed disease until the questionnaires at 2 years of age. Chronic diseases included congenital heart disease, congenital renal and urinary disease, neurofibromatosis, tuberous sclerosis, and hypothyroidism. The height, weight, measurement season, and age of the children and BP were measured simultaneously. When any covariates were missed, we excluded the child from our analysis. Furthermore, we excluded children if the height deviated more or less than five standard deviations (SDs) from the Japanese standard growth chart [22].

### Analytic plan

We excluded children with either missing or unstable BP measurements and covariates to estimate the standardized BP reference table. We then described the participants’ background based on gestational weeks, birth weight, height, and weight at BP measurement, measurement seasons, systolic BP (SBP), and DBP. The mean and SD were calculated for continuous variables, and the number of cases and proportion in the entire target population were calculated for categorical variables.

Next, we separated the SBP and DBP values and analyzed each BP using two methods for making BP reference values: the polynomial regression model and the lambda-mu-sigma (LMS) model. In the polynomial regression model, based on the previous study in the USA [13], the analysis was estimated by sex, age (years), and height Z score (Zht). However, in the present study, the age of the children was approximately 2 years. We estimated the analysis using only sex and Zht and excluded children < 24 months of age. Then, according to the previous study, we estimated the expected BP values using sex, Zht, squared Zht, cubed Zht, and quartet Zht as each SBP and DBP. The Zht was calculated based on standard Japanese data [22]. The observed fit curve, residual density, and normal Q–Q were plotted to confirm the fit of the estimated models.

Third, we estimated the reference BP values by the LMS method. The LMS model was developed for each sex with the height Z score as the explanatory variable in a generalized additive model using the LMS method [23]. The LMS method should indicate a more appropriate estimation when each BP distribution was not equal to each Zht. The parameters L, M, and S were set to Box–Cox power, median, and coefficient of variance, and the combination with the minimum Bayesian information criteria was selected for each equivalent degree of freedom. The observed fit curve, residual density, and normal Q–Q were plotted to confirm the fit of the estimated models.

Fourth, for making the reference tables, on applying the Zht, corresponding to the 5th, 10th, 25th, 50th, 75th, 90th, and 95th percentile points to SBP and DBP percentile curves, respectively, the expected BP values corresponding to each percentile point for the height and BP were obtained by each model. Both the polynomial regression and LMS models retrospectively estimated similar values for SBP and DBP and found similar trends in the observed fit curve, residual density, and normal Q–Q plot; however, the LMS model had a better fit of approximately two SD ranges of height graphically (Supplementary Figures S1 and S2). The coefficients of each model are listed in Supplementary Tables S2 and S3.

We used R statistical software, version 3.6.3 (The R Foundation for Statistical Computing, Vienna, Austria) for statistical analyses.

## Results

### Participants’ background

**Fig. 1:**
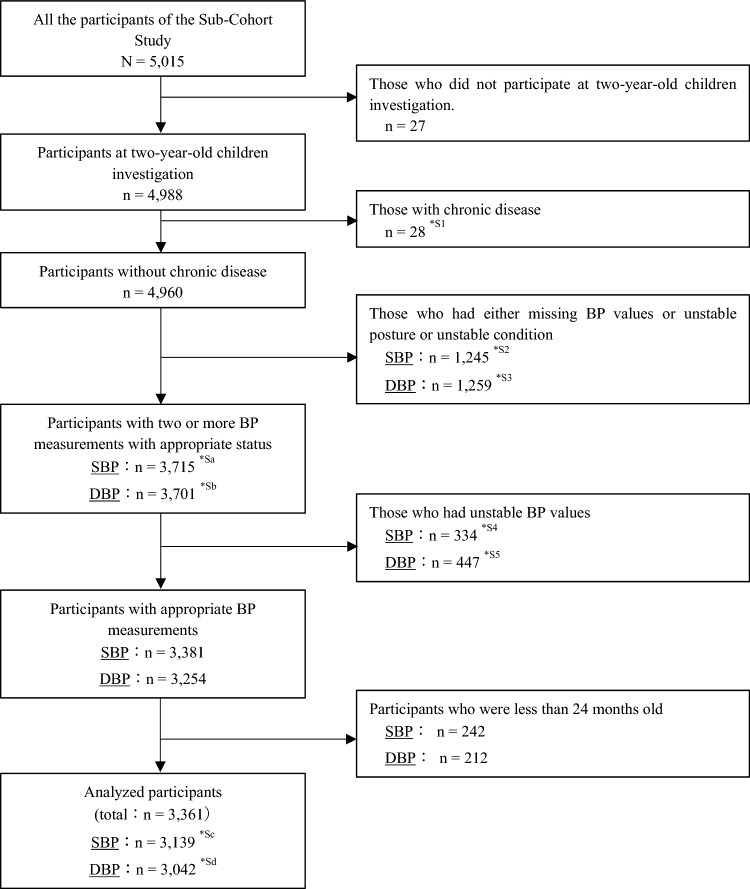
Flowcharts for selecting participants for SBP and DBP analysis. *SBP* systolic blood pressure, *DBP* diastolic blood pressure, *BP* blood pressure, *S1–*S5 the detailed contents and number of each item are shown in Supplementary Table S4; *Sa-*Sd the detailed breakdowns of each number are shown in Supplementary Table S4

Of the 5,015 participants in the Sub-Cohort Study, 4988 were included in this study. The SBP and DBP of 3139 and 3042 participants (3361 in total) were adapted for our analysis. Among the excluded children, 28 had a chronic disease. In the SBP and DBP groups, 1256 and 1259 had either missing BP values or unstable measurement or unstable condition, 334 and 447 were measured with unstable BP values, and 242 and 212 were under 2 years of age, respectively (Fig. 1.) Detailed explanations for each factor are shown in Supplementary Table S4.

Of those 3361 children, 1672 (49.7%) were boys and 144 (4.3%) had preterm birth. The winter measurements were less than those in the other seasons (Table 1 and Supplementary Table S5). Supplementary Table S6 lists the age in months at the time of the BP measurement.

**Table 1:** Participants’ demographic background

Name of regional center	*n*	Sex	Height	Weight	SBP			DBP	
		Male	(cm)	(kg)	Available	(mmHg)	Available		(mmHg)
Hokkaido	207	90 (43.5%)	83.9 (2.9)	11.5 (1.2)	195 (94.2%)	88.5 (8.7)	181 (87.4%)		51.6 (7.8)
Miyagi	295	149 (50.5%)	84.6 (2.9)	11.8 (1.2)	277 (93.9%)	92.0 (6.4)	265 (89.8%)		52.8 (7.8)
Fukushima	404	198 (49.0%)	84.1 (2.8)	11.6 (1.1)	375 (92.8%)	95.4 (7.8)	359 (88.9%)		53.6 (8.7)
Chiba	158	65 (41.1%)	83.7 (2.7)	11.5 (1.2)	141 (89.2%)	93.1 (9.5)	140 (88.6%)		51.7 (8.6)
Kanagawa	184	88 (47.8%)	84.1 (3.4)	11.6 (1.4)	172 (93.5%)	93.0 (7.5)	169 (91.8%)		54.0 (7.5)
Koshin	264	135 (51.1%)	83.7 (2.7)	11.4 (1.1)	249 (94.3%)	88.4 (7.7)	241 (91.3%)		53.4 (6.2)
Toyama	203	112 (55.2%)	84.8 (3.0)	11.6 (1.2)	172 (84.7%)	88.2 (8.7)	177 (87.2%)		52.2 (8.0)
Aichi	177	83 (46.9%)	83.9 (2.9)	11.3 (1.1)	161 (91.0%)	86.7 (8.8)	156 (88.1%)		45.4 (8.0)
Kyoto	155	80 (51.6%)	84.5 (3.0)	11.8 (1.2)	151 (97.4%)	87.4 (7.1)	142 (91.6%)		48.4 (7.1)
Osaka	326	156 (47.9%)	83.9 (3.1)	11.4 (1.2)	321 (98.5%)	88.9 (5.8)	320 (98.2%)		47.5 (5.8)
Hyogo	201	122 (60.7%)	84.1 (3.1)	11.3 (1.2)	197 (98.0%)	91.3 (6.8)	194 (96.5%)		51.8 (6.6)
Tottori	98	44 (44.9%)	83.6 (2.8)	11.2 (1.0)	93 (94.9%)	82.1 (6.2)	93 (94.9%)		51.3 (5.5)
Kochi	223	114 (51.1%)	83.5 (2.7)	11.5 (1.1)	200 (89.7%)	92.9 (7.2)	187 (83.9%)		55.8 (7.7)
Fukuoka	277	140 (50.5%)	83.8 (2.6)	11.4 (1.2)	262 (94.6%)	88.9 (6.3)	258 (93.1%)		51.2 (6.8)
Minamikyushu/Okinawa	189	96 (50.8%)	83.5 (2.7)	11.5 (1.1)	173 (91.5%)	95.0 (8.8)	160 (84.7%)		52.9 (8.5)
Total	3361	1672 (49.7%)	84.0 (2.9)	11.5 (1.2)	3139 (93.4%)	90.6 (8.1)	3042 (90.5%)		51.7 (7.9)

Data are presented as mean (SD) or *n* (%)

*SBP* systolic blood pressure, *DBP* diastolic blood pressure

### The estimated reference BP values

In Table 2, we described the estimated BP values by the LMS method. For 2-year-old children with heights in the 50th percentile, the 50th, 90th, 95th, and 99th percentile reference values of systolic BP (mmHg) for boys were 91, 102, 106, and 112, and that for girls were 90, 101, 103, and 109 for girls, respectively, and those of DBP for boys were 52, 62, 65, and 71, respectively, and 52, 62, 65, and 71 for girls, respectively. The estimated BP values by the polynomial regression model are shown in Supplementary Table S7.

**Table 2:** Reference BP values by LMS method

Age (year)	Sex	BP percentile	Systolic BP (mmHg)							Diastolic BP (mmHg)						
			Percentile of height^a^							Percentile of height^a^						
			5th	10th	25th	50th	75th	90th	95th	5th	10th	25th	50th	75th	90th	95th
2	Boys	50th	90	90	91	91	92	92	92	51	51	51	52	52	53	53
		90th	101	101	102	102	103	103	104	61	61	62	62	63	63	63
		95th	105	105	105	106	106	107	107	64	64	65	65	66	66	66
		99th	111	111	112	112	113	113	114	70	70	70	71	71	72	72
	Girls	50th	89	89	90	90	91	91	92	51	51	51	52	52	53	53
		90th	99	99	100	101	101	102	102	61	61	62	62	63	63	64
		95th	102	102	103	103	104	105	105	64	64	65	65	66	66	67
		99th	107	108	108	109	110	110	111	69	70	70	71	72	72	73

*BP* blood pressure, *LMS method* lambda-mu-sigma method

^a^The *Z* score height values converted to their corresponding percentile values

## Discussion

In the present study, we created reference BP values for 2-year-old Japanese children based on a detailed survey of the JECS. These are the first measurements by auscultatory methods from a sufficient number of Japanese children nationwide.

Selecting an appropriate survey group is essential to create a reliable reference BP value. The appropriate reference BP value for each race or ethnic group must be created based on their respective data. Previous studies have shown that BP values differ between urban and rural areas [24] and vary based on temperature and season [25]. As shown in Table 1 and Supplementary Table S5, the detailed survey data of JECS were collected year-round at 15 Regional Centers across Japan. The participants of the detailed survey of the JECS were considered appropriate for creating reference BP values for Japanese children.

A proper BP measurement device is indispensable for BP measurements. We used aneroid sphygmomanometer to measure the BP. Applying an aneroid sphygmomanometer to measure BP was considered acceptable for the following reasons. First, the gold standard method for measuring BP is auscultation using a mercury sphygmomanometer, and the reference BP values for US children [11, 13], which are the most widely used values worldwide, are based on BP values measured by this method. However, since mercury sphygmomanometers can no longer be used due to their negative environmental impact, aneroid sphygmomanometers are widely used in clinical practice. Second, the difference between BP readings by an aneroid and mercury sphygmomanometer has been reported to be very small [26], and the use of either device is recommended by the AAP guidelines [11].

An appropriate measurement technique is critical for the BP measurements used to create reference BP values. The technique used in our study was based on the AAP guidelines [11] and was considered reliable. Additionally, to obtain appropriate BP values, it is important not only to calm the participants during measurements but also to select which measurement to use from multiple available measurements. Each participant was measured thrice, and the average value of two consecutive measurements with a difference of < 5 mmHg was adopted as the BP value. Comparing this selection method with those of previous major guidelines [11, 12, 20, 21] (Supplementary Table S8) our method was considered capable of selecting stable BP values similar to the other methods.

Differences in reference DBP values were observed between the present study and US guidelines [11]. Taking the 50th percentile reference BP values for 2-year-old children with heights in the 50th percentile as an example, the SBP (mmHg) for Japanese boys and girls were 91 and 90, and those for the US boys and girls were 89 and 89, respectively, which were almost the same in Japan and the USA. The DBP (mmHg) was 52 for both boys and girls in Japan. However, DBP for US boys and girls were 44 and 48, which were 8 and 4 mmHg lower, respectively, compared with Japanese children. Although the reference BP values created in the present study differed from those of the US guidelines, our reference BP values were sufficiently reliable because our reference BP values were measured and aggregated in a standardized and appropriate manner based on a sufficient number of Japanese children.

Differences were observed between the reference BP values obtained using aneroid manometers in this study and those using oscillometric devices in the previous study. In the report on the reference BP values of Japanese children using an automated BP recorder (Dinamap Model 8104), the 95th percentile BP values for 2-year-olds were 115/69 mmHg for boys and 121/70 mmHg for girls [27]. In the present study, the 95th percentile BP values for 2-year-olds at the 50th percentile height were 106/65 mmHg for boys and 103/65 mmHg for girls, with a large difference in SBP. These differences are possibly attributed to variations in BP measurement methods, devices, BP value selections, and study participants.

A reliable analysis model is required to create the reference BP values. We used LMS and polynomial regression models for the analysis, both of which were considered valid in this study. The reference BP values for US children [13] were created using a polynomial regression model. The LMS method has been used in the previously reported BP reference for children [14–16]. In the drawing of the residual density plot and the normal Q-Q plot, which were performed to confirm the fit of each estimated regression model, both models generally followed a normal distribution (Supplementary Fig. S1 and S2). The difference in the expected BP values estimated by each model was small.

The LMS model could more accurately estimate the reference BP value in this study as it was considered more valid for a wider range of Zht than the polynomial regression model.

This study has some limitations. First, we analyzed BP measurements in 2-year-olds. Further study is needed to create the reference BP values for older children, as the data after 2 years will be aggregated from now on. Second, not all participants were exactly 24 months old when BP was measured (Supplementary Table S6). Although most participants were slightly more than 2 years at the time of BP measurement, the measurements from children with a small age range were summarized as the data for 2 years. Third, the differences in mean measured BP values at each of the 15 Regional Centers were observed up to 13.3 (range 82.1–95.4) mmHg for SBP and 10.4 (range 45.4–55.8) mmHg for DBP. In JECS, the BP measurement methods were standardized. Biases due to examiners' skills and noise, such as anxiety from measurement environment conditions for each child, are unavoidable in clinical settings; it is meaningful to create reference values while considering these biases. Further investigation is required to determine whether there are regional differences in blood pressure.

Fourth, although BP values may vary depending on whether an individual adopts the supine and sitting positions, the differences could not be assessed, as the number of participants in the supine position was only eight among all participants. Therefore, the BP values for children who cannot adopt sitting positions need to be evaluated individually. Finally, an aneroid sphygmomanometer requires regular semiannual maintenance [26]. JECS does not stipulate that the maintenance interval of sphygmomanometers is to be performed semiannually. However, they are inspected visually before each BP measurement. JECS only uses newly acquired sphygmomanometers within the 2-year period.

## Conclusion

The reference BP values for 2-year-old Japanese children were created based on auscultatory BP measurements from the JECS’s detailed survey using the LMS method and are now available for routine practice.

## Supplementary Information

Below is the link to the electronic supplementary material.

Supplementary file1 (DOCX 2703 KB)

## Data Availability

Data are unsuitable for public deposition due to ethical restrictions and the legal framework of Japan. It is prohibited by the Act on the Protection of Personal Information (Act No. 57 of May 30, 2003, Amendment September 9, 2015) to publicly deposit data containing personal information. Ethical Guidelines for Epidemiological Research enforced by Japan’s Ministry of Education, Culture, Sports, Science and Technology and Ministry of Health, Labour and Welfare also restrict the open sharing of epidemiologic data. All inquiries about access to data should be sent to: jecs-en@nies.go.jp. The person responsible for handling enquiries sent to this e-mail address is Dr. Shoji F. Nakayama, JECS Programme Office, National Institute for Environmental Studies.
