# Supplementary material for: Reference blood pressure values obtained using the auscultation method for 2-year-old Japanese children: from the Japan Environment and Children’s Study
Source: Clin Exp Nephrol. 2023 Jun 30;27(10):857–64. doi: 10.1007/s10157-023-02370-w (PMC10504195; doi:10.1007/s10157-023-02370-w)
Supplement: Supplementary file 1 — Supplementary file1 (DOCX 2703 KB) [file 10157_2023_2370_MOESM1_ESM.docx]

**Electronic Supplementary Material**

**Clinical and Experimental Nephrology**

**Reference blood pressure values obtained using the auscultation method for two-year-old Japanese children: From the Japan Environment and Children’s Study**

Naoya Fujita^1^, Hidetoshi Mezawa^2^, Kyongsun Pak^3^, Osamu Uemura^4^, Kiwako Yamamoto-Hanada^2^, Miori Sato^2^, Mayako Saito-Abe^2^, Yumiko Miyaji^2^, Limin Yang^2^, Minaho Nishizato^2^, Yukihiro Ohya^2^, Kenji Ishikura^5^, Yuko Hamasaki^6^, Tomoyuki Sakai^7^, Kazuna Yamamoto^7^, Shuichi Ito^8^, Masataka Honda^9^, Yoshimitsu Gotoh^10^ and the Japan Environment and Children’s Study Group*

^1^Department of Pediatric Nephrology, Aichi Children’s Health and Medical Center, 426 7-chome, Morioka-cho, Obu, Aichi 474-8710 Japan

^2^Medical Support Center for Japan Environment and Children’s Study, National Center for Child Health and Development, 2-10-1 Okura, Setagaya-ku, Tokyo, 157-8535, Japan

^3^Division of Biostatistics, Department of Data Management, Center for Clinical Research and Development, National Center for Child Health and Development, 2-10-1 Okura, Setagaya-ku, Tokyo, 157-8535, Japan

^4^Department of Pediatrics, Ichinomiya Medical Treatment & Habilitation Center, 1679-2 Tomida-nagaresuji, Ichinomiya-city, Aichi, 494-0018, Japan

^5^Department of Pediatrics, Kitasato University School of Medicine, 1-15-1 Kitazato, Minami-Ku, Sagamihara, Kanagawa 252-0374 Japan

^6^Department of Nephrology, Toho University Faculty of Medicine, 6-11-1 Omori Nishi, Ota-ku, Tokyo, 143-8541, Japan

^7^Department of Pediatrics, Shiga University of Medical Science, Tsukinowa, Seta, Otsu, Shiga, 520-2192, Japan

^8^Department of Pediatrics, Graduate School of Medicine, Yokohama City University, 3-9 Fukuura, Kanazawa-ku, Yokohama, 236-0004 Japan

^9^Department of Pediatric Nephrology, Tokyo Metropolitan Children's Medical Center, 2-8-29 Musashidai, Fuchu, Tokyo, 183-8561 Japan

^10^Department of Pediatric Nephrology, Japanese Red Cross Aichi Medical Center Nagoya Daini Hospital, 2‐9 Myoken‐cho Showa‐ku Nagoya‐shi Aichi 466‐8650 Japan

*Study group members are listed in the Appendix.

The e-mail address, telephone, and fax numbers of the corresponding author:

Naoya Fujita

E-mail: fujita708@hkg.odn.ne.jp

TEL: +81-562-43-0500

FAX: +81-562-43-0513

**Supplementary Table S1**. Methods used to measure BP in the detailed survey in JECS

| 1. Participants should rest for approximately 5 minutes, such as by watching a DVD to calm themselves, and then nurses or doctors should measure their BP. |
| --- |
| 1. BP should be measured by auscultation using an aneroid sphygmomanometer. The width of the bladder of the cuff should be more than 40% of the upper arm’s circumference, and its length should be at least 80%. The ratio of the width and length of the cuff’s bladder should be more than 1:2. |
| 1. Blood pressure may be measured in a sitting, supine, or other position, but care should be taken to prevent the participants from crying. The posture at the time of measurement should be recorded. |
| 1. BP should be measured with the right arm, with the elbow at heart level. |
| 1. The phase I Korotkoff and phase V Korotkoff sounds should be taken as SBP and DBP. When the Korotkoff sounds are heard to 0 mmHg, BP should be measured again, and if the result is the same, the phase IV Korotkoff sounds should be taken as DBP. |
| 1. BP should be measured three times. Approximately one minute after the end of the first measurement, the second measurement is obtained. Similarly, the third is obtained approximately one minute after the second measurement. |
| 1. The measured BP values and status of the participants during each measurement (resting, crying, with body movement, sleeping) should be recorded on the designated recording sheet. |

BP: blood pressure; JECS: the Japan Environment and Children’s Study; DVD: Digital Versatile Disc; SBP: systolic blood pressure; DBP: diastolic blood pressure

**Supplementary Table S2.** Estimated values for each variable of the LMS method

|  | Systolic BP | | Diastolic BP | |
| --- | --- | --- | --- | --- |
| Variable Name | Boys | Girls | Boys | Girls |
| Equivalence of degrees of freedom of L, M and S | 1, 2, 1 | 1, 2, 1 | 1, 2, 1 | 1, 2, 1 |
| Bayesian information criteria | 10,993.1 | 11,051.6 | 10,474.2 | 10,726.7 |
| Normalized Height (:X) |  |  |  |  |
| Coefficients of L(Box-Cox power) | 0.11423 | 0.78716 | 0.82538 | 0.65121 |
| Formula of L | ~Intercept | ~Intercept | ~Intercept | ~Intercept |
| Coefficients of M(Median) | 91.21576, 0.63364 | 90.34111, 0.79798 | 51.83832, 0.58679 | 51.85951, 0.75926 |
| Formula of M | ~Intercept + β*X | ~Intercept + β*X | ~Intercept + β*X | ~Intercept + β*X |
| Coefficients of S(Coefficient of variance) | -2.39864 | -2.44227 | -1.87139 | -1.89506 |
| Formula of S | ~Intercept | ~Intercept | ~Intercept | ~Intercept |
| n (persons) | 1,553 | 1,586 | 1,497 | 1,545 |

LMS method: Lambda-Mu-Sigma method. BP: blood pressure.

**Supplementary Table S3.** Estimated values for each value of the polynomial regression method

|  |  | Systolic BP | | Diastolic BP | |
| --- | --- | --- | --- | --- | --- |
| Variable Name | Symbol | Boys | Girls | Boys | Girls |
| Intercept | α | 91.47719 | 90.38598 | 51.77788 | 52.23970 |
| Normalized Height |  |  |  |  |  |
| Zht | γ_1 | 0.47381 | 0.79778 | 0.04915 | 0.72435 |
| Zht^2 | γ_2 | -0.02239 | 0.01580 | 0.07299 | -0.34954 |
| Zht^3 | γ_3 | 0.11978 | 0.05206 | 0.25633 | -0.02546 |
| Zht^4 | γ_4 | 0.05015 | 0.02461 | 0.04626 | 0.04316 |
| Standard deviation | σ | 8.3245 | 7.8535 | 7.9503 | 7.7695 |
| n (persons) |  | 1,553 | 1,586 | 1,497 | 1,545 |

BP: blood pressure.

**Supplementary Table S4**. Detailed explanations for the flow chart (Fig.1)

(a) *S1. Number of participants excluded due to comorbidities

| Total number | 28 |
| --- | --- |
| Tetralogy of Fallot | 1 |
| Complete transposition of the great arteries and double outlet right ventricle | 1 |
| Hydronephrosis | 13 |
| Cystic kidney disease | 1 |
| Single kidney | 2 |
| Bladder exstrophy and Cloacal exstrophy | 2 |
| Hydronephrosis and single kidney | 1 |
| Congenital hypothyroidism | 7 |

(b) *S2. Number of participants excluded for SBP due to their state at the time of measurement, the number of measurements, and physical findings

| Total number (with duplication) | 1,256 |
| --- | --- |
| Posture at the time of BP measurement: other than sitting or supine position, or missing data. | 527 |
| The appearance of the participants during BP measurement: crying, sleeping, or missing data: |  |
| During the first BP measurement | 776 |
| During the second BP measurement | 854 |
| During the third BP measurement | 873 |
| SBP data: |  |
| Missing in all three of three measurements | 323 |
| Missing in two of three measurements | 174 |
| Sex: missing data | 0 |
| Body height: |  |
| Missing data | 527 |
| Boys with a height of less than 70.4 cm or more than 100.4 cm | 0 |
| Girls with a height of less than 69.8 cm or more than 93.0 cm | 5 |
| Body temperature: more than 38.0℃ | 17 |

SBP: systolic blood pressure; BP: blood pressure.

(c) *S3. Number of participants excluded for DBP due to their state at the time of measurement, the number of measurements, and physical findings

| Total number (with duplication) | 1,259 |
| --- | --- |
| Posture at the time of BP measurement: other than sitting or supine position, or missing data. | 527 |
| The appearance of the participants during BP measurement: crying, sleeping, or missing data: |  |
| During the first BP measurement | 778 |
| During the second BP measurement | 855 |
| During the third BP measurement | 877 |
| DBP data: |  |
| Missing in three of three measurements | 336 |
| Missing in two of three measurements | 180 |
| Zero reading on DBP measurement: |  |
| The first DBP measurement | 4 |
| The second DBP measurement | 7 |
| The third DBP measurement | 4 |
| Sex: Missing data | 0 |
| Body height: |  |
| Missing data | 527 |
| Boys with a height of less than 70.4 cm or more than 100.4 cm | 0 |
| Girls with a height of less than 69.8 cm or more than 93.0 cm | 5 |
| Body temperature: more than 38.0℃ | 17 |

DBP: diastolic blood pressure; BP: blood pressure.

(d) *S4. Number of SBP readings excluded due to the difference between two consecutive values exceeding 5 mmHg.

| Total number | 334 |
| --- | --- |
| Participants with the following two SBP measurements out of three and the difference between these two values exceeded 5 mmHg: |  |
| The first and the second measurement. | 65 |
| The second and the third measurement. | 27 |
| The first and the third measurement**. | 6 |
| Participants with three SBP measurements and the difference between the first and the second, and the second and the third measurements both exceeded 5 mmHg. | 236 |

SBP: systolic blood pressure.

** The case where only the first and the third measurement were performed was regarded as continuous measurement.

(e) *S5. Number of DBP readings excluded due to the difference between two consecutive values exceeding 5 mmHg.

| Total number | 447 |
| --- | --- |
| Participants with the following two DBP measurements out of three and the difference between these two values exceeded 5 mmHg: |  |
| The first and the second measurement. | 100 |
| The second and the third measurement. | 32 |
| The first and the third measurement**. | 14 |
| Participants with three DBP measurements and the difference between the first and the second, and the second and the third measurements both exceeded 5 mmHg. | 301 |

DBP: diastolic blood pressure.

** The case where only the first and the third measurement were performed was regarded as continuous measurement.

(f) *Sa. Participants with two or more SBP measurements

| Total number | 3,715 |
| --- | --- |
| Participants who obtained two SBP measurements | 487 |
| Participants who obtained three SBP measurements | 3,228 |

SBP: systolic blood pressure.

(g) *Sb. Participants with two or more DBP measurements

| Total number | 3,701 |
| --- | --- |
| Participants who obtained two DBP measurements | 491 |
| Participants who obtained three DBP measurements | 3,210 |

DBP: diastolic blood pressure.

(h) *Sc. Body posture of the participants during SBP measurements

| Total number | 3,139 |
| --- | --- |
| Sitting position | 3,131 |
| Supine position | 8 |

SBP: systolic blood pressure.

(i) *Sd. Body posture of the participants during DBP measurements

| Total number | 3,042 |
| --- | --- |
| Sitting position | 3,034 |
| Supine position | 8 |

DBP: diastolic blood pressure.

**Supplementary Table S5**. Participants’ demographic background

|  |  | At Birth | | At the Time of BP Measurement | | | |
| --- | --- | --- | --- | --- | --- | --- | --- |
|  | n | Gestational Weeks | Birth Weight | Season | | | |
| Name of Regional Center |  | <37 w | <2,500 g | Spring | Summer | Autumn | Winter |
| Hokkaido | 207 | 8 (3.9%) | 15 (7.2%) | 48 (23.2%) | 59 (28.5%) | 74 (35.7%) | 26 (12.6%) |
| Miyagi | 295 | 11 (3.7%) | 24 (8.1%) | 71 (24.1%) | 97 (32.9%) | 79 (26.8%) | 48 (16.3%) |
| Fukushima | 404 | 23 (5.7%) | 34 (8.4%) | 96 (23.8%) | 132 (32.7%) | 121 (30.0%) | 55 (13.6%) |
| Chiba | 158 | 6 (3.8%) | 11 (7.0%) | 30 (19.0%) | 51 (32.3%) | 51 (32.3%) | 26 (16.5%) |
| Kanagawa | 184 | 4 (2.2%) | 14 (7.6%) | 48 (26.1%) | 47 (25.5%) | 55 (29.9%) | 34 (18.5%) |
| Koshin | 264 | 6 (2.3%) | 21 (8.0%) | 54 (20.5%) | 73 (27.7%) | 85 (32.2%) | 52 (19.7%) |
| Toyama | 203 | 7 (3.4%) | 15 (7.4%) | 45 (22.2%) | 59 (29.1%) | 68 (33.5%) | 31 (15.3%) |
| Aichi | 177 | 10 (5.6%) | 13 (7.3%) | 36 (20.3%) | 63 (35.6%) | 54 (30.5%) | 24 (13.6%) |
| Kyoto | 155 | 6 (3.9%) | 9 (5.8%) | 24 (15.5%) | 52 (33.5%) | 48 (31.0%) | 31 (20.0%) |
| Osaka | 326 | 22 (6.7%) | 40 (12.3%) | 75 (23.0%) | 98 (30.1%) | 98 (30.1%) | 55 (16.9%) |
| Hyogo | 201 | 8 (4.0%) | 15 (7.5%) | 35 (17.4%) | 76 (37.8%) | 56 (27.9%) | 34 (16.9%) |
| Tottori | 98 | 2 (2.0%) | 9 (9.2%) | 19 (19.4%) | 28 (28.6%) | 29 (29.6%) | 22 (22.4%) |
| Kochi | 223 | 10 (4.5%) | 17 (7.6%) | 43 (19.3%) | 65 (29.1%) | 86 (38.6%) | 29 (13.0%) |
| Fukuoka | 277 | 7 (2.5%) | 11 (4.0%) | 60 (21.7%) | 90 (32.5%) | 76 (27.4%) | 51 (18.4%) |
| Minamikyushu/Okinawa | 189 | 14 (7.4%) | 18 (9.5%) | 38 (20.1%) | 57 (30.2%) | 64 (33.9%) | 30 (15.9%) |
| Total | 3,361 | 144 (4.3%) | 266 (7.9%) | 722 (21.5%) | 1,047 (31.2%) | 1,044 (31.1%) | 548 (16.3%) |

Data are presented as mean (SD) or n (%)

SBP: systolic blood pressure; DBP: diastolic blood pressure.

**Supplementary Table S6.** Age in months at the time of BP measurement

| Age (m) | Number (n) | (%) |
| --- | --- | --- |
| 24 | 1,261 | 37.5 |
| 25 | 1,383 | 41.2 |
| 26 | 530 | 15.8 |
| 27 | 168 | 5.0 |
| 28 | 13 | 0.4 |
| 29 | 2 | 0.1 |
| 30 | 3 | 0.1 |
| 31 | 1 | 0.0 |
| Total | 3,361 | 100.0 |

BP: blood pressure.

**Supplementary Table S7.** BP percentile table by polynomial regression method

| Age (Year) | Sex | BP percentile | Systolic BP (mmHg) | | | | | | |  | Diastolic BP (mmHg) | | | | | | |
| --- | --- | --- | --- | --- | --- | --- | --- | --- | --- | --- | --- | --- | --- | --- | --- | --- | --- |
|  |  |  | Percentile of Height | | | | | | |  | Percentile of Height | | | | | | |
|  |  |  | 5^th^ | 10^th^ | 25^th^ | 50^th^ | 75^th^ | 90^th^ | 95^th^ |  | 5^th^ | 10^th^ | 25^th^ | 50^th^ | 75^th^ | 90^th^ | 95^th^ |
| 2 | boys | 50^th^ | 90 | 91 | 91 | 91 | 92 | 92 | 93 |  | 51 | 51 | 52 | 52 | 52 | 53 | 54 |
|  |  | 90^th^ | 101 | 101 | 102 | 102 | 103 | 103 | 104 |  | 61 | 62 | 62 | 62 | 62 | 63 | 64 |
|  |  | 95^th^ | 104 | 104 | 105 | 105 | 106 | 106 | 107 |  | 64 | 64 | 65 | 65 | 65 | 66 | 67 |
|  |  | 99^th^ | 110 | 110 | 110 | 111 | 111 | 112 | 112 |  | 70 | 70 | 70 | 70 | 70 | 71 | 72 |
|  |  |  |  |  |  |  |  |  |  |  |  |  |  |  |  |  |  |
|  | girls | 50^th^ | 89 | 89 | 90 | 90 | 91 | 92 | 92 |  | 51 | 51 | 52 | 52 | 53 | 53 | 53 |
|  |  | 90^th^ | 99 | 99 | 100 | 100 | 101 | 102 | 102 |  | 60 | 61 | 62 | 62 | 63 | 63 | 63 |
|  |  | 95^th^ | 102 | 102 | 103 | 103 | 104 | 105 | 105 |  | 63 | 64 | 64 | 65 | 65 | 65 | 65 |
|  |  | 99^th^ | 107 | 108 | 108 | 109 | 109 | 110 | 110 |  | 69 | 69 | 70 | 70 | 71 | 71 | 71 |

BP: blood pressure

**Supplementary Table S8.** Number of BP measurements and calculation methods for determining the values to be adopted as the BP value in major guidelines

|  | JSH 2019 [21] | AAP 2017 [11] | ACC/AHA 2017 [20] | ESH 2016 [12] |
| --- | --- | --- | --- | --- |
| Number of BP measurement | Multiple times | Twice | Two times or more | Three times |
| Calculation method for average BP value | Average of two stable* BP measurements | Average of two BP measurements | Average of two or more BP measurements | Average of the last two BP measurements |

* The difference is < 5 mmHg as a guide

BP: blood pressure.

JSH 2019: The Japanese Society of Hypertension Guidelines for the Management of Hypertension.

AAP 2017: Clinical Practice Guideline for Screening and Management of High Blood Pressure in Children and Adolescents (by the American Academy of Pediatrics).

ACC/AHA 2017: 2017 ACC/AHA/AAPA/ABC/ACPM/AGS/AphA/ASH/ASPC/NMA/PCNA Guideline for the Prevention, Detection, Evaluation, and Management of High Blood Pressure in Adults: Executive Summary: A Report of the American College of Cardiology/American Heart Association Task Force on Clinical Practice Guidelines.

ESH 2016: 2016 European Society of Hypertension guidelines for the management of high blood pressure in children and adolescents.

**Supplementary Fig S1.** Fit curve of the observed SBP and LMS models, residual density plot, and normal Q-Q plot of the LMS model of SBP for boys (a). SBP for girls (b), DBP for boys (c), and DBP for girls (d). In the fit curve, the solid, long-dashed, short-dashed, and dotted lines indicate 50^th^, 90^th^, 95^th^, and 99^th^ percentiles, respectively. SBP: systolic blood pressure; LMS model: Lambda-Mu-sigma model; DBP: diastolic blood pressure.


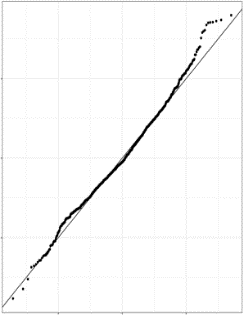

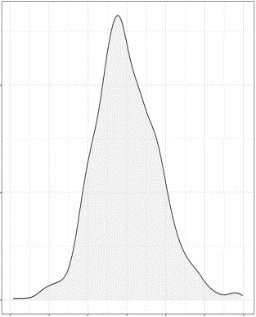

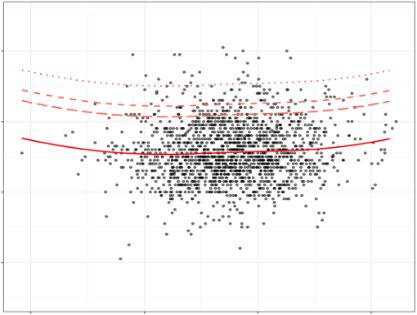


Density

0.04

0.02

Residual

-30

-20

20

0

0.00

SBP (mmHg)

120

100

80

60

Z score for height

-4

-2

2

0

Standardized Residuals

2

0

-2

Theoretical Quantiles

-2

2

0

Observed SBP and polynomial regression model

Residual density plot

Normal Q-Q plot

**(a)**

-10

10

30


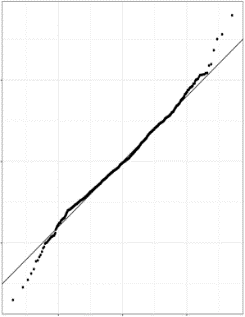

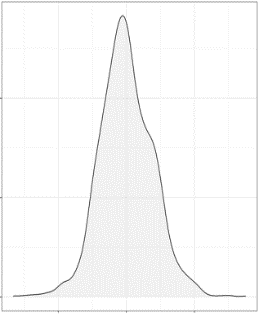

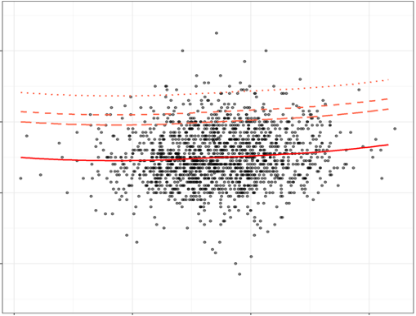


Density

Residual

SBP (mmHg)

120

100

80

60

Z score for height

-4

-2

2

0

Standardized Residuals

2.5

0.0

-2.5

Theoretical Quantiles

-2

2

0

**(b)**

0.04

0.02

0.00

-20

0

20


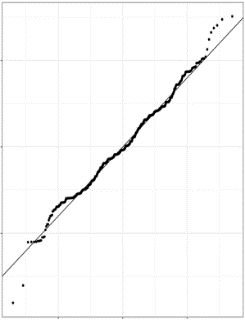

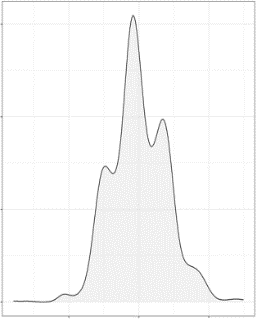

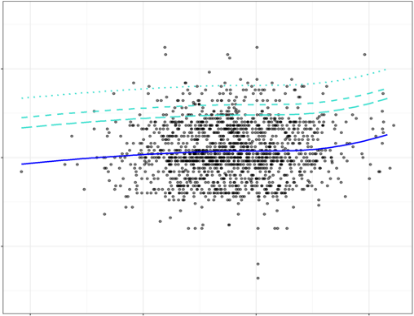


Density

0.04

0.02

Residual

0.00

DBP (mmHg)

75

50

25

Z score for height

-4

-2

2

0

Standardized Residuals

2.5

0.0

-2.5

-5.0

Theoretical Quantiles

-2

2

0

**(c)**

0.06

-20

0

20


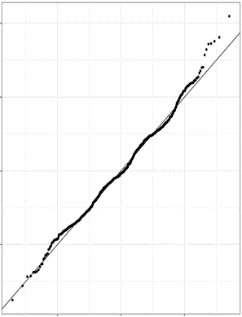

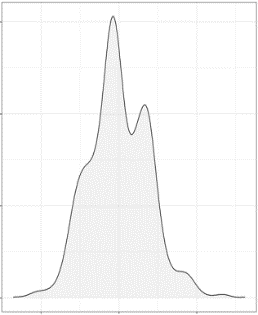

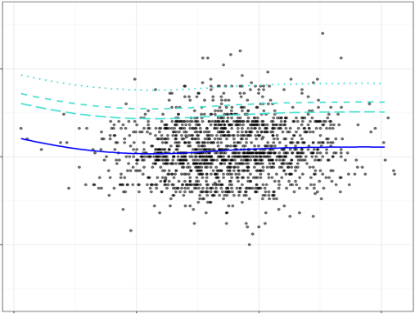


Density

Residual

DBP (mmHg)

75

50

25

Z score for height

-4

-2

2

0

Standardized Residuals

2

0

-2

Theoretical Quantiles

-2

2

0

(d)

4

-4

0.04

0.02

0.00

0.06

-20

0

20

**Supplementary Fig S2.** Fit curve of the observed SBP and polynomial regression model, residual density plot, and normal Q-Q plot of the polynomial regression model of the SBP for boys (a), SBP for girls (b), DBP for boys (c), and DBP for girls (d). In the fit curve, the solid, long-dashed, short-dashed, and dotted lines indicate 50^th^, 90^th^, 95^th^, and 99^th^ percentiles, respectively. SBP: systolic blood pressure; DBP: diastolic blood pressure.

**Supplementary Method: The JECS main study and JECS sub-cohort study** [18, 19]

In the JECS main study, the participant mothers answered questionnaires twice during pregnancy, one month after delivery, after answering a questionnaire every six months. Moreover, the JECS research coordinators gathered maternal and infant medical information by medical record transcription at mid-pregnancy, birth, and one month after birth.

The Sub-Cohort Study had the following eligibility criteria: 1) the children born after April 1, 2013; 2) complete questionnaire data and medical record transcripts of children and their mothers from the first trimester to 6 months of age; 3) biospecimens (except for umbilical cord blood) of children and their mothers collected during the first trimester, second-third trimester, and delivery.

Participants who met the eligibility criteria of the Sub-Cohort Study were randomly recruited, and the recruitment rate was 48.6%. From April 2015 to January 2017, the Sub-Cohort Study was conducted at two years of age. In the Sub-Cohort Study for two-year-olds, all the participants had face-to-face investigations, such as anthropometric measurements, BP measurements, and developmental testing between 23 and 27 months of age.
